# The potential of cell sheet technique on the development of hepatocellular carcinoma in rat models

**DOI:** 10.1371/journal.pone.0184004

**Published:** 2017-08-29

**Authors:** Alaa T. Alshareeda, Katsuhisa Sakaguchi, Mohammed Abumaree, Nur Khatijah Mohd Zin, Tatsuya Shimizu

**Affiliations:** 1 Stem Cells and Regenerative Medicine Department, King Abdullah International Medical Research Center, King Abdulaziz Medical City, Ministry of National Guard Health Affairs, Riyadh, Kingdom of Saudi Arabia; 2 Institute of Advanced Biomedical Engineering and Science, Tokyo Women Medical University, Tokyo, Japan; 3 School of science and engineering, Waseda University, Tokyo, Japan; 4 College of Science and Health Professions, King Saud bin Abdulaziz University for Health Sciences, King Abdulaziz Medical City, Ministry of National Guard Health Affairs, Riyadh, Kingdom of Saudi Arabia; EFS, FRANCE

## Abstract

**Background:**

Hepatocellular carcinoma (HCC) is considered the 3rd leading cause of death by cancer worldwide with the majority of patients were diagnosed in the late stages. Currently, there is no effective therapy. The selection of an animal model that mimics human cancer is essential for the identification of prognostic/predictive markers, candidate genes underlying cancer induction and the examination of factors that may influence the response of cancers to therapeutic agents and regimens. In this study, we developed a HCC nude rat models using cell sheet and examined the effect of human stromal cells (SCs) on the development of the HCC model and on different liver parameters such as albumin and urea.

**Methods:**

Transplanted cell sheet for HCC rat models was fabricated using thermo-responsive culture dishes. The effect of human umbilical cord mesenchymal stromal cells (UC-MSCs) and human bone marrow mesenchymal stromal cells (BM-MSCs) on the developed tumour was tested. Furthermore, development of tumour and detection of the liver parameter was studied. Additionally, angiogenesis assay was performed using Matrigel.

**Results:**

HepG2 cells requires five days to form a complete cell sheet while HepG2 co-cultured with UC-MSCs or BM-MSCs took only three days. The tumour developed within 4 weeks after transplantation of the HCC sheet on the liver of nude rats. Both UC-MSCs and BM-MSCs improved the secretion of liver parameters by increasing the secretion of albumin and urea. Comparatively, the UC-MSCs were more effective than BM-MSCs, but unlike BM-MSCs, UC-MSCs prevented liver tumour formation and the tube formation of HCC.

**Conclusions:**

Since this is a novel study to induce liver tumour in rats using hepatocellular carcinoma sheet and stromal cells, the data obtained suggest that cell sheet is a fast and easy technique to develop HCC models as well as UC-MSCs have therapeutic potential for liver diseases. Additionally, the data procured indicates that stromal cells enhanced the fabrication of HepG2 cell sheets. This provides the foundation for future research using stromal cells in preclinical and clinical investigations.

## Introduction

Hepatocellular carcinoma (HCC) is the most prevalent primary liver malignancy and the sixth most common cancer worldwide with poor prognosis. While the number of HCC’s cases is on the increase, [1] they are commonly diagnosed in the late stages [2]. There are various factors that increase the risk of HCC, such are hepatitis B and C viruses (chronic infections), liver cirrhosis in alcoholics, foods contaminated with aflatoxins, as well as exposure to chemical carcinogens [3–5]. Since HCC is a heterogeneous cancer with a variety of genetic abnormalities, there are assorted molecular pathways in the regulation of proliferation and necrobiosis [6].

Considering the physiological and genetic resemblance between rodents and humans, the potential of xenograft implantation, the accessibility of gene targeting methods, the short lifespan in addition to the capacity of breeding, rodents are usually preferred for cancer research [7, 8]. In previous studies, mouse models have been genetically modified to imitate pathophysiological and molecular features of HCC to evaluate the influence of oncogenes either alone, in combination with other oncogenes or carcinogenic agents [9]. Cancer cell lines are used extensively to investigate the biology of cancer as well as to study the hypotheses in translational research and the cell lines relevance depending on the proximate resemblance to the tumours that are being investigated. The aim of this study is to develop a HCC animal model using cell sheet technology using HepG2 as it is the most widely used cell line [10, 11]. Bin Chen et al [12] have stated that HepG2 showed the highest correlation to HCC tumours in comparison to other cell lines. The most commonly utilised approach to induce cancer in animals is by injecting cancer cell suspension. Though it is a clear and convenient approach, there are several disadvantages. Of such, in order to generate cell suspension, an enzymatic digestion is needed to harvest the cancer cells, as a result, this method would lose a certain amount of adhesion proteins in which will then result in the reduction of efficiency of engraftment of the transplanted cells. Consequently, there will be no guarantee for the transplanted cell to form cancerous tissues. Another issue connected to this method is the difficulty to control the size of the tumour. Additionally, another serious hitch in this method is the rapid transition of the transplanted cancer cell to non-targeted organs by not only but including the blood circulation system [13]. Previously, a myriad of chemicals capable of developing liver cancer has been investigated in several animal models namely dogs [14], Mongolian gerbils [15], monkeys [16], rats [17], and mice [18, 19]. These models were essential in the premalignant and malignant liver lesions studies allowing a validated conclusion for the bio-pathology mechanisms of the underlying human liver tumour and also to assess new therapeutic strategies.

Circa 1999, a novel method of tissue engineering based on cell sheet engineering was established in Okano’s Lab in Japan [20, 21]. This technique utilises the thermo-responsive culture surface which allows reversible adhesion/detachment of cells by controlling the hydrophobicity of the surface. This method offers a more gentle way of harvesting intact 3D cultured cells (cell sheet) that maintains the deposited extracellular matrix (ECM) as well as cell-cell interactions [22]. Thence, the poor cell survival as a result of dissociated cell suspension injection could be significantly improved [23].

Hypervascularity is one of the significant features of HCC, however, this condition varies significantly during the different stages of HCC [13]. Vascular support plays a key role in the growth of a tumour, as vasculatures are important to provide a continuous supply of essential nutrients to the tumour [14]. Hereof, angiogenesis holds a crucial part in different homeostatic processes and non-neoplastic diseases. Thus, inhibition of neoangiogenesis is projected to be a promising therapeutic approach for HCC. In a previous study by Muto, Shirabe et al recorded that several pharmacological drugs could efficiently target angiogenesis and a number of proteins with anti-angiogenic activities has been identified for HCC[15]. However, in a contradicting study by Gauthier and Ho, patients with late stage HCC has very poor survival rate [16]. Therefore, the discovery of alternative therapy that could result for a more effective treatment of HCC is paramount.

Thus, the current known first line therapy for HCC cases are; ablative treatments, surgical resection and allogeneic transplantation [17, 18]. However, these surgical interventions are not advisable for patients at an advanced stage as well as patients with poor liver functions [19]. Furthermore, according to Ota K., Teraoka S. et al., there are limitations for liver transplantation especially in its availability and it is most suitable for patients with earlier stages of HCC [20]. Hence at the present, it is utmost crucial for the development of an effective general therapy for patients with advanced stages of HCC.

Adequately, the potential of mesenchymal stromal cells (MSCs) to treat liver diseases are also under investigation. A number of experimental and clinical studies suggest that MSC transplantation can sustain liver function in chronic and acute liver injury [21–24]. The potential for stem cell based therapy is being progressively investigated in the field of cancers. Though the discovery is not without its drawbacks, MSCs have been presented to have both pro-tumourigenic and anti-tumourigenic activities. However, this highly depends on several factors such as the source and type of MSCs, types of tumour cells, condition of the of experiment in vivo and in vitro, soluble molecules secreted by MSCs as well as the interactions of MSCs with the host’s immune cells or cancer cells. Taking for example, UC-MSCs could cause brain tumour regression dissimilar to adipose tissue derived MSCs (AT-MSCs) [25, 26]. In another instance, BMMSCs was shown to increase the tumour’s growth in models of gastric, breast and ovarian cancers [27–29]. In another instance, BM-MSCs was shown to increase the tumour’s growth in models of gastric, breast and ovarian cancers [30].

## Materials and methods

### Cell culture

Cell lines of liver hepatocellular carcinoma (HepG2, PromoCell, USA), human bone marrow mesenchymal stromal cells (BM-MSCs) (PromoCell, USA), human umbilical cord mesenchymal stromal cells (UC-MSCs) (PromoCell, USA) were cultured in a culture medium consists of high glucose DMEM (Sigma, Tokyo, Japan), 10% fetal bovine serum (FBS) (CCB, NICHIREI BIOSCIENCE, Tokyo, Japan) and 1% penicillin/streptomycin (Life Technology, Carlsbad, CA, USA) while human umbilical vein endothelial cells (HUVECs) (Lonza, USA) were cultured in endothelial cell culture medium (EBM) (EGM, Lonza, Tokyo, Japan) containing 2% FBS and vascular endothelial growth factor (VEGF). Cells were then incubated at 37°C in a humidified atmosphere containing 5% CO_2_ and 95% air.

### Tube network formation assay

For tube network formation, growth factor reduced Matrigel (#354234, Corning, Tokyo, Japan) was thawed overnight on ice at 4 ^o^C and 200 μl was then added to a 24-well plate and incubated at 37°C as indicated above. The next day, HepG2 cells were then cultured alone or with HUVECs BM-MSCs or UC-MSCs at different HepG2/ HUVECs or BM-MSCs or UC-MSCs ratios ranging 1:1, 3:1, 1:3, and 1:5, where the ration 1 is 250x10^3^ cells in 24-well plate containing DMEM culture medium supplemented with 50ng/ml vascular endothelial growth factor (VEGF, R&D system, Tokyo, Japan). HUVECs were cultured alone as a positive control. Following 14 hours incubation as indicated above, the tube network was observed under Nikon Eclipse Ti fluorescence microscope and imaged at x4 magnification using Axio Vision 4.8 software. Three independent experiments were performed for every set in this study.

### Tube network staining with endothelial marker CD31 and nucleus staining Hoechst 33258

To stain cells with endothelial marker (CD31) and nucleus marker (Hoechst 33258), cells were fixed with 4% paraformaldehyde for 30 min at room temperature (RT) and were then washed for three times with sterile PBS. Followed by blocking with One Histo blocking solution (nacalai tesque, Tokyo, Japan) for 1 h at RT, cells were again washed with PBS for three times and incubated with primary antibody (Anti-human CD31 monoclonal antibody, Clone #JC70A, Dako, Tokyo, Japan) diluted for 100x in PBS for 2 h at RT. Cells were then washed with PBS for three times and later incubated with secondary antibody (Anti-mouse IgG conjugated with Alexa Fluor, clone #A11017, Life Technology, Tokyo, Japan) diluted for 100x in PBS. Later followed by incubation for 2 h at RT, cells were then again washed with PBS for three times and stained with nucleus marker (Hoechst 33258; 1:500 PBS) for 5 min at RT. Staining was then observed and images were taken as described above using Axio Vision 4.8 software.

### ELISA for VEGF determination

In order to investigate VEGF level in vitro, the conditioned medium (CM) of three different groups were collected in day one and day 7 of culture- (incubation at 37^0^ C with 4% CO2)- and then Human VEGF Quantikine ELISA Kit from R&D was used. Group 1; 1.25 x10^6^ of HUVEC cells (as positive control), group 2; the co-cultured of HepG2 (1x10^6^) with UC-MSC (250x10^3^) and group 3; co-cultured of HepG2 (1x10^6^) with BM-MSC (250x10^3^). Optical density was read at 450nm on BIO RAD 680 Microplate Reader. All cells were cultured on 100 mm^2^ polystyrene culture surface dish (Falcon 3002, Becton Dickinson, Tokyo, Japan). Each experement was done three times.

### Urea and albumin quantification

Urea and albumin levels were measured in the CM of HepG2 or HepG2 co-cultured with UC-MSCs or BM-MSCs by quantitative colorimetric and ELISA, respectively.The number of the cells and the condition of (co)-cultivation were similar to VEGF ELISA test. The levels of Urea and albumin level were measured on days 1 and 7. For urea measurement, urea assay kit (DIUR-100, BioAssay System, Tokyo, Japan) was used following the manufacturer’s instructions. For albumin measurement, albumin ELISA test kit (Human albumin ELISA Quantification Set, Bethyl Laboratories Inc., Tokyo, Japan) was used following the manufacturer’s instructions. Optical density was read at 430nm on BIO RAD 680 Microplate Reader. Each experiment was performed in triplicate. Three independent experiments were performed in this study.

### Cell sheet construction

Cell sheets were prepared in mono layer according to previously published technique [31]. Briefly, 3.5 cm temperature-responsive UpCell culture dishes (type D, Sigma, Tokyo, Japan) were coated with undiluted FBS and incubated for 24 h at 37°C as indicated above. Coating dishes with FBS supports the growth of cells in a monolayer and prevents the aggregation of cells [31]. After removing the excess FBS, 1x10^6^ HepG2 cells alone or with 250x10^3^ UC-MSCs or BM-MSCs were seeded onto dishes containing DMEM culture medium and cultured at 37°C as indicated above. To detach sheets of co-cultured cells, dishes were incubated for 30–40 min at 20°C while the dishes of HepG2 sheets incubated for 1 h at RT. The cell sheets were detached spontaneously and floated into the aqueous media. Immediately after detachment, the entire monolayer cell sheet with media was gently aspirated into the tip of a 10 ml pippette and transferred onto 60 mm2 polystyrene culture surface dish (Falcon 3002, Becton Dickinson, Tokyo, Japan). After placement, media was dropped onto the center of the sheet to spread folded parts of the transferred cell sheets. After spreading the cell sheet, media was then aspirated to adhere the cell sheet to the culture surface. Following incubation for 30 min at 37°C as indicated above, the cell sheets were recultured and used in subsequent experiments. Cell sheets were observed and images were taken as described above. Image J software was used to measure the surface area of the cell sheets, a ruler was also used. Each experiment was performed in triplicate. Three independent experiments were performed in this study.

### Staining of actin fiber

Cell sheets were fixed in 37% formaldehyde for 30 min at RT and were then permeabilised with Triton X-100 for 10 min at RT. Following incubation with 1 g phalloidin Alexa Fluor 436 Phalloidin (Molecular Probes™, Invetrogen, Tokyo, Japan), the sheets were washed with PBS and observed under Nikon Eclipse Ti fluorescence microscope and imaged at x100 magnification using Axio Vision 4.8 software.

### Animal

All the experimental protocols for animals were approved by the Institutional Animal Care and Use Committee of Tokyo Women’s Medical University (#AE16-58). 4–8 weeks old male nude rats (*F344/NJcl-rnu/rnu*; Japan Laboratory Animals, Inc, Japan) were used in the study. Rats were housed in polypropylene cages lined with husk in standard environmental conditions (temperature 25±2°C, relative humidity 55±10% and 12:12 light: dark cycle). Rats were fed on a standard pellet diet and had free access to water. All animal experiments were performed according to the guidelines of Tokyo Women’s Medical University on animal use, and consistent with the guidelines for the care and use of laboratory animals prepared by the Institute of Laboratory Animal Resources (ILAR). In this study, four groups of rats were used and each group consists of 5 rats. Group A (Negative control): non-transplanted rats, Group B (Positive control): rats transplanted with cell sheet constructed from HepG2 cells, Group C (Test 1): rats transplanted with cell sheet constructed from HepG2 and UC-MSCs, Group D (Test 2): rates transplanted with cell sheet constructed from HepG2 and human BM-MSCs. The sample size was calculated using online sample size calculator [32].

### Cell sheet transplantation

**Fig 1:**
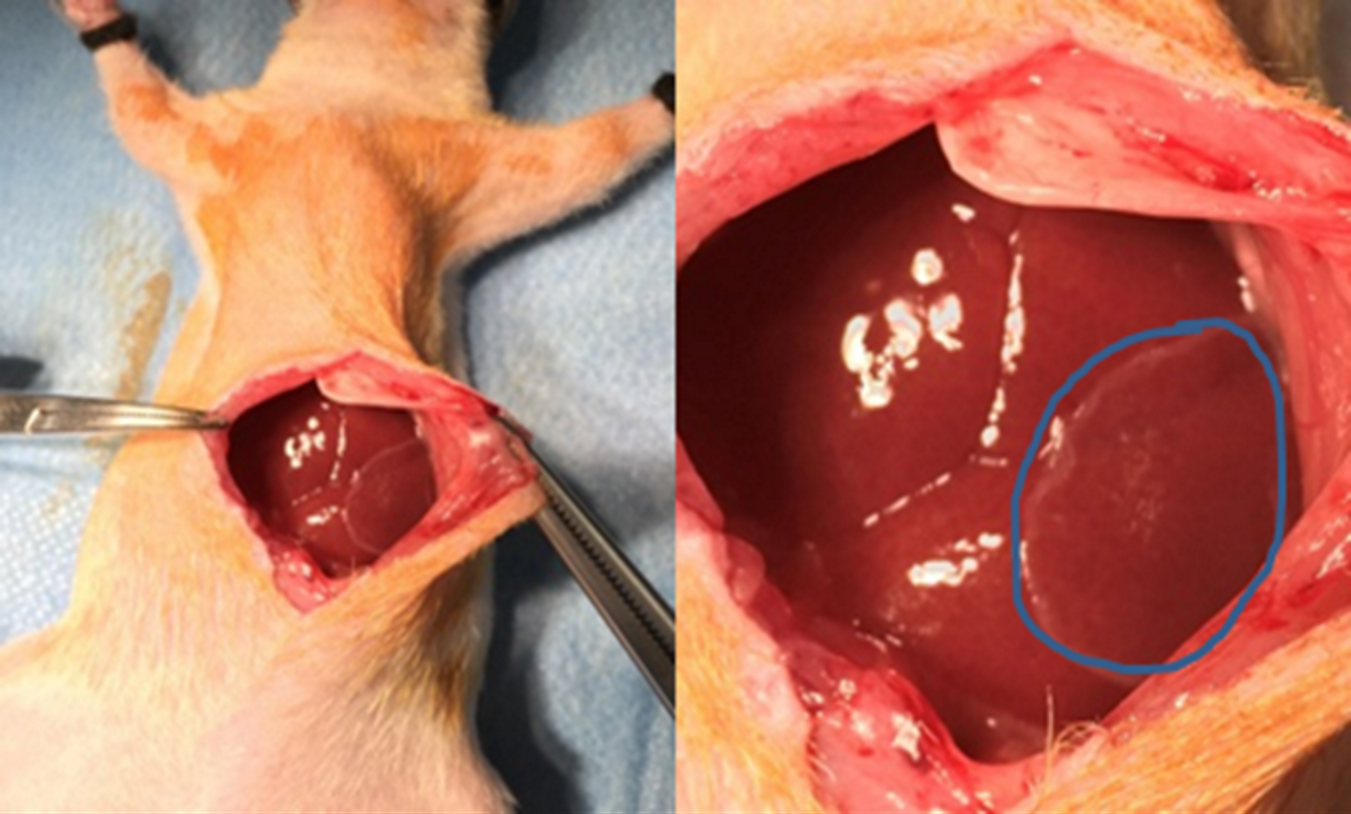
Image of the transplantation procedure of cell sheets on rat’s liver. The cell sheet was transplanted onto one site of the liver surface of each rat using a support membrane to facilitate handling. The circle shows the transplanted cell sheet on the liver.

Cell sheets were detached as described above and washed with Hanks’ solution prior to surgery. Rats were then anesthetised with 1.5–3.8% isoflurane (Pfizer, Tokyo, Japan) via nose cones and a cut between the skin and the underlying muscles was then done. Following the separation of the skin from the underlying muscles, muscle was cut an approximately 7–15 cm in size and a cell sheet was transplanted onto one site of the liver tissue (Fig 1) with the assessment of a polypropylene supporting membrane (6×4 cm), which was used to facilitate the handling of cell sheet. The membrane was then removed slowly after the attachment of the cell sheet on the liver. Skin incisions were then closed with 5.0 nylon sutures. After recovery from anaesthesia, the behavioural signs of postoperative pain, post-surgical infections, and the surgical incision site were monitored. The rats were monitored daily for palpable tumours for 4 weeks after transplantation. Post-surgery, all the animals were observed daily for any sign of illness, injury, physical deficit or abnormal behaviour and again the incision was also observed. At week one, two, three and four after transplantation, the rats were anesthetised as previously described and 100–300 μl of blood samples were then collected from the tail of rats and immediately kept for 30–45 min on ice. Serum were collected by centrifugation at 3,000 rpm for 15 min at 4°C and stored at -20°C and serum albumin was estimated using Bromocresol Green (MAK124 Sigma, Sigma Aldrich, Country) while urea was estimated as described above. After one month of transplantation, rats were sacrificed by exsanguination under anaesthesia with 1.5–3.8% isoflurane (Pfizer, Tokyo, Japan) via nose cones and the transplanted areas were opened and liver tissues were then resected for histological and immunohistochemical analysis. The size of the tumours on the liver were measured using a clipper.

### Histological and immunohistochemial analysis

For cross-sectional observations, liver tissues adhered to cell sheets were fixed with 4% paraformaldehyde for 48 h at 4 ^o^C. Tissues were then dehydrated through a series of graded ethanol solution 70% and 100%. Following, immersing in xylene, tissues were embedded in paraffin wax using a tissue processor (Geno staff CT-Pro20, Tokyo, Japan) and sliced into sections of 5μm in thickness. Hematoxylin and eosin (H&E) staining was performed by conventional method. Each section was stained for three times.

To stain tissues with a tumour marker GPC3 (Novous, Tokyo, Japan), immunohistochemical staining was performed using universal ABC kit (VECTASTAIN kit, Tokyo, Japan). Briefly, 5μm sections from paraffin-embedded tissue blocks were de-paraffinized, rehydrated in xylenes and graded alcohol series. Following antigen retrieval using Dako target retrieval antigen solution (Dako, Tokyo, Japan) in a pressure cooker (Dako cytomation, Japan) for 20 min, the sections were incubated with blocking solution (Blocking One Histo, nacalai tesque, Tokyo, Japan) for 30 min at RT and then washed with PBS. Sections were then incubated with anti-mouse GPC3 monoclonal antibody (Novous, Tokyo, Japan) for 1h at RT. After washing with PBS, sections were incubated with biotinylated secondary antibody solution (Biotinylated Universal anti-Mouse IgG/Rabbit IgG, VECTASTAIN kit, Tokyo, Japan) for 30 min at RT, and then washed with PBS. Sections were then incubated for 5 min with VECTASTAIN® *elite* ABC Reagent and washed with PBS. Following staining with 3,3-diaminobenzidine substrate–chromogen solution (Dako, Tokyo, Japan), sections were washed with de-ionized water, immersed in haematoxylin nuclear stain (Dako, Tokyo, Japan) for 5 min, and then washed with tap water and coverslips mounted with MOUNT-QUICK (HistoTec™, Tokyo, Japan).

### Quantification of GPC3

The numbers of positive cells were quantified manually by counting the number of GPC3-only stained tumour cells in 10 random power fields (20×). The percentage of tumour cells was estimated on a blind basis at least three times independently. Only the cancer cells were counted. The results on the level of GPC3 expression was presented as H-scoring scale from 0 to 300; the carcinoma with GPC3 staining intensity (optical density [OD] of staining; range between 0:negative, 1:weak, 2:medium, and 3:strong) multiplied by the percentage (%) of carcinoma with GPC3 staining.

### Statistical analysis

Data are expressed as mean±SD. An unpaired Student’s *t* test was performed to compare 2 groups. Tow-way ANOVA was used for multiple group comparison. A value of *P*<0.05 was considered significant.

## Results

### Tube network formation in vitro

**Fig 2:**
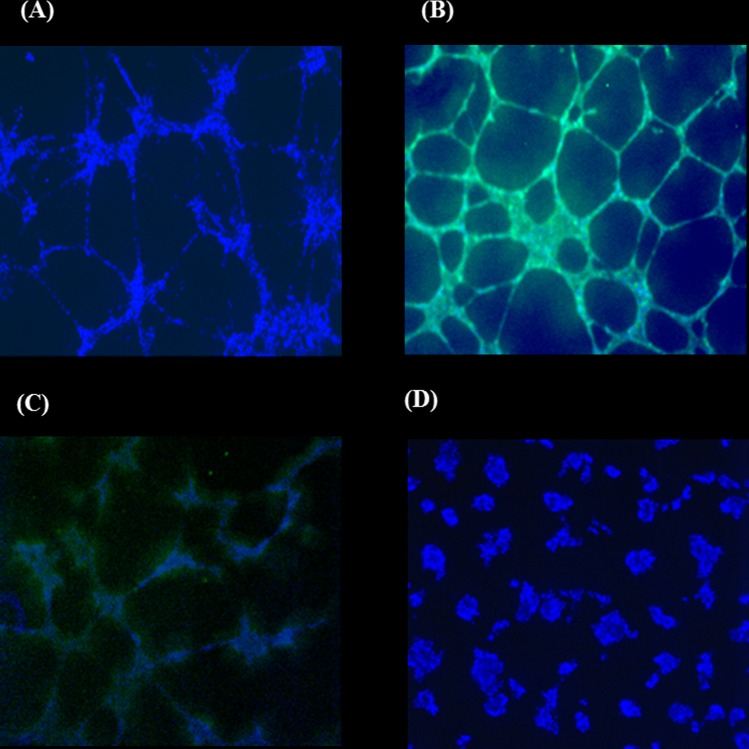
Vascularisation of HepG2 and MSCs on Matrigel after 14h incubation. (A) HepG2, (B)HUVECs, (C) BM-MSC, (D) UC-MSC. Cells were seeded into Matrigel in the presence of VEGF. CD31 (green) and Hoechst 33258 staining (blue). x4 magnification.

To assess the ability of cells to form tube network in vitro, human UC-MSCs, and human BM-MSCs, HepG2 and HUVECs were seeded on Matrigel. After 14 h of incubation, HepG2 cells showed a complete tube network formation (Fig 2A). As expected the positive control HUVECs also formed a complete network (Fig 2B). BM-MSCs too were able to form tube networks (Fig 2C) unlike UC-MSCs (Fig 2D).

### UC-MSCs prevent HepG2 tube network formation in vitro

**Fig 3:**
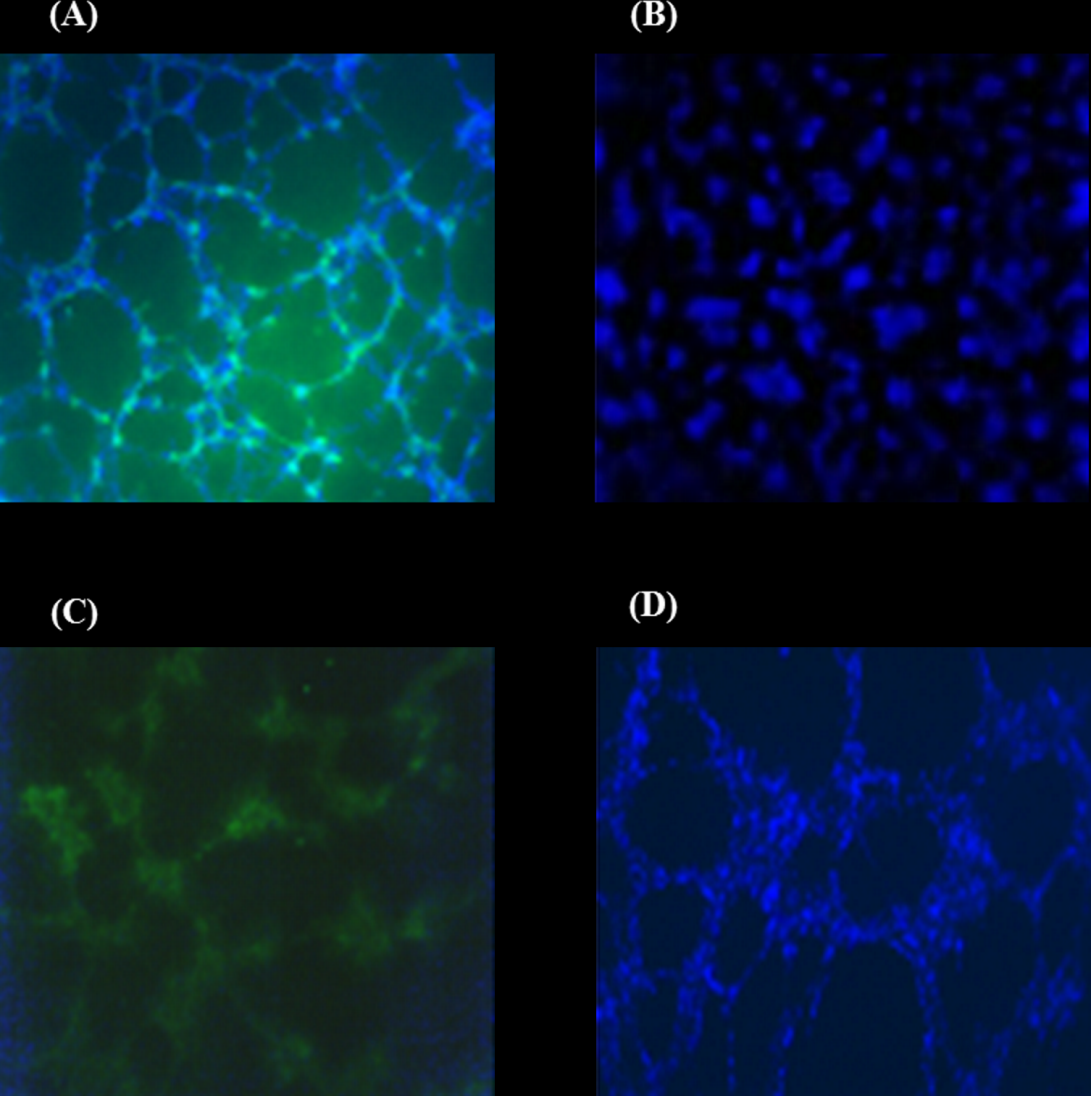
Vascularisation of co-culture of HUVEC or MSCs with HepG2 on Matrigel after 14h incubation. (A) HUVEC+HepG2, (B) UC-MSC+HepG2, (C&D) BM-MSC+HepG2. Cells were seeded into Matrigel in the presence of VEGF. Tubes started to form after 14 hours. CD31 (green) and Hoechst 33258 staining (blue). The presented ratio of the co-culture cells is 1:1. 4x magnification.

To determine whether SCs can modulate the network formation of HepG2 cells in vitro, HepG2 cells were co-cultured with SCs cells on Matrigel and tube formation was then assessed. All the cells were stained with the endothelial cell marker CD31and Hoechst staining to detect the viability of the cells. The tube network was not affected when HepG2 cells were co-cultured with HUVECs and CD 31marker was positive in the network (Fig 3A). On the contrary, the tube network formation of HepG2 was completely inhibited by UC-MSCs (Fig 3B), therefore, HepG2 with UC-MSCs showed a negative staining for CD31(Fig 3B). The addition of BM-MSCs to HepG2 culture had no effect on forming tube network, hence the CD31 marker was positive in this network (Fig 3C& D). The viability of the cells was confirmed by a positive staining of blue Hoechst for all the cultures (Fig 3).

**Fig 4:**
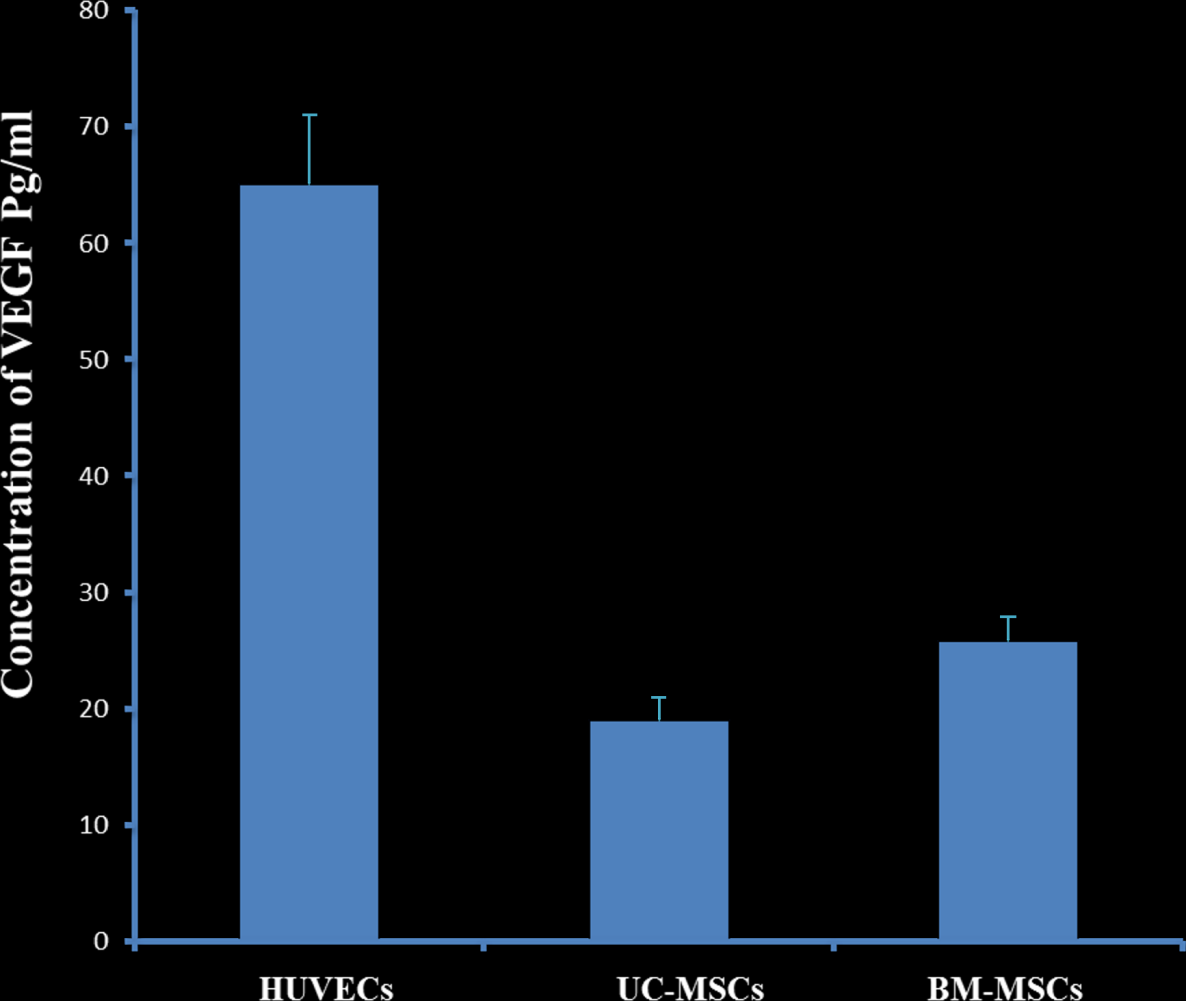
Human VEGF concentration of co-cultured HepG2 with MSCs (UC or BM) after one week of incubation. HUVECs were used as a positive control. Tow-way NOVA (P = 0.001).

VEGF level was investigated in both stromal cells; BM-MSC and UC-MSC and HUVECs was used as a positive control using ELISA. There was a statistical significance between the three groups (Fig 4, P = 0.001), where BM-MSC showed a higher level of VEGF than UC-MSC, whereas, HUVECs was the highest.

### Human stromal cells effect on the liver parameters of HepG2 cancer cell line in vitro

**Fig 5:**
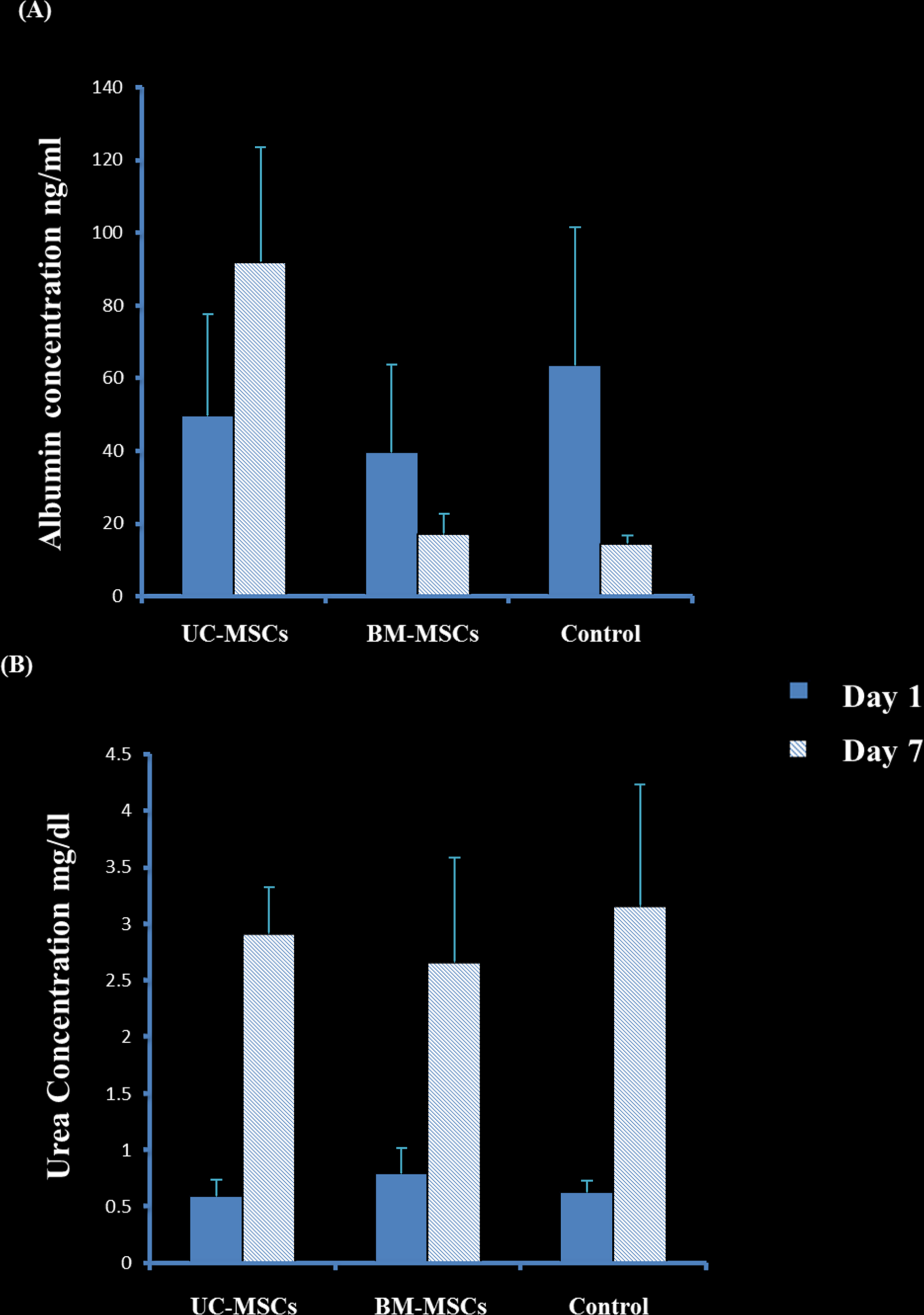
Urea and albumin concentration of cell culture of HepG2 and stromal cells at day 1 and day 7 day. Where control is only HepG2 cells, whereas each stromal cells bar represents co-cultured cells of HepG2 and MSC (UC or BM). At day 7 in the culture, the secretion of albumin by the co-culture of HepG2 and UC-SCs significantly increased as compared to untreated HepG2, (P = 0.03). For urea, after 7 days all the cultured cells showed an increase in the urea level within 7 days (P<0.05). Mean± SD. Tow-way ANOVA.

To evaluate whether the co-culture of HepG2 cells with MSC (BM-MSC and UC-MSC) would affect the secretion of albumin or urea of HepG2 in vitro, the level of albumin and urea was quantified in the CM obtained from the co-culture of HepG2 with SCs by ELISA and colorimetric assay, respectively. The secretion of albumin by the co-culture of HepG2 and UC-MSCs significantly increased at day 7 in culture as compared to day 1 while the co-culture of HepG2 and BM-MSCs secretion of albumin dropped to the half at day 7 as compared to day 1, but was not significant, P>0.05 (Fig 5A). Additionally, at day 7 in culture, the secretion of albumin by the co-culture of HepG2 and UC-MSCs significantly increased as compared to the control (untreated HepG2) P = 0.03 and the co-culture of HepG2 and BM-MSCs, P<0.05 (Fig 5A). In contrast, at day 7 in culture, the secretion of albumin by the co-culture of HepG2 and human BM-MSCs was not changed as compared to untreated HepG2, P>0.05 (Fig 5A).

The secretion of urea increases substantially after 7 days within all the groups: HepG2 co-cultured with UC-MSCs or BM-MSCs or by HepG2 alone but does not differ significantly between the groups (P>0.05, Fig 5B), however, there was a statistical significance between day 1 and day 7 within each group (P<0.05, Fig 5B).

### Cell sheet construction and characteristics

Cell sheet was constructed to examine the effect of SCs on the fabrication of HepG2 cell sheets. Untreated HepG2 required five days to form a complete cell sheet while three days were required to form a cell sheet using HepG2 co-cultured with UC-MSCs or BM-MSCs. This indicates that human MSCs enhances the fabrication of HepG2 cell sheets. Furthermore, the detachment of the HepG2 cell sheets with no residual cells associated with its surface required to lower the temperature from 37°C to 20°C for 1 h while the dishes of HepG2 co-cultured with UC-MSCs or BM-MSCs were incubated at the same temperature as HepG2 cell sheet for only 30 min. This further confirms that MSCs can improve the fabrication of HepG2 sheets. In addition, HepG2 cell sheets were fragile but the addition of UC-MSCs or BM-MSCs to HepG2 strengthened the sheets. This also confirms the ability of MSCs to improve the fabrication of HepG2 sheets.

**Fig 6:**
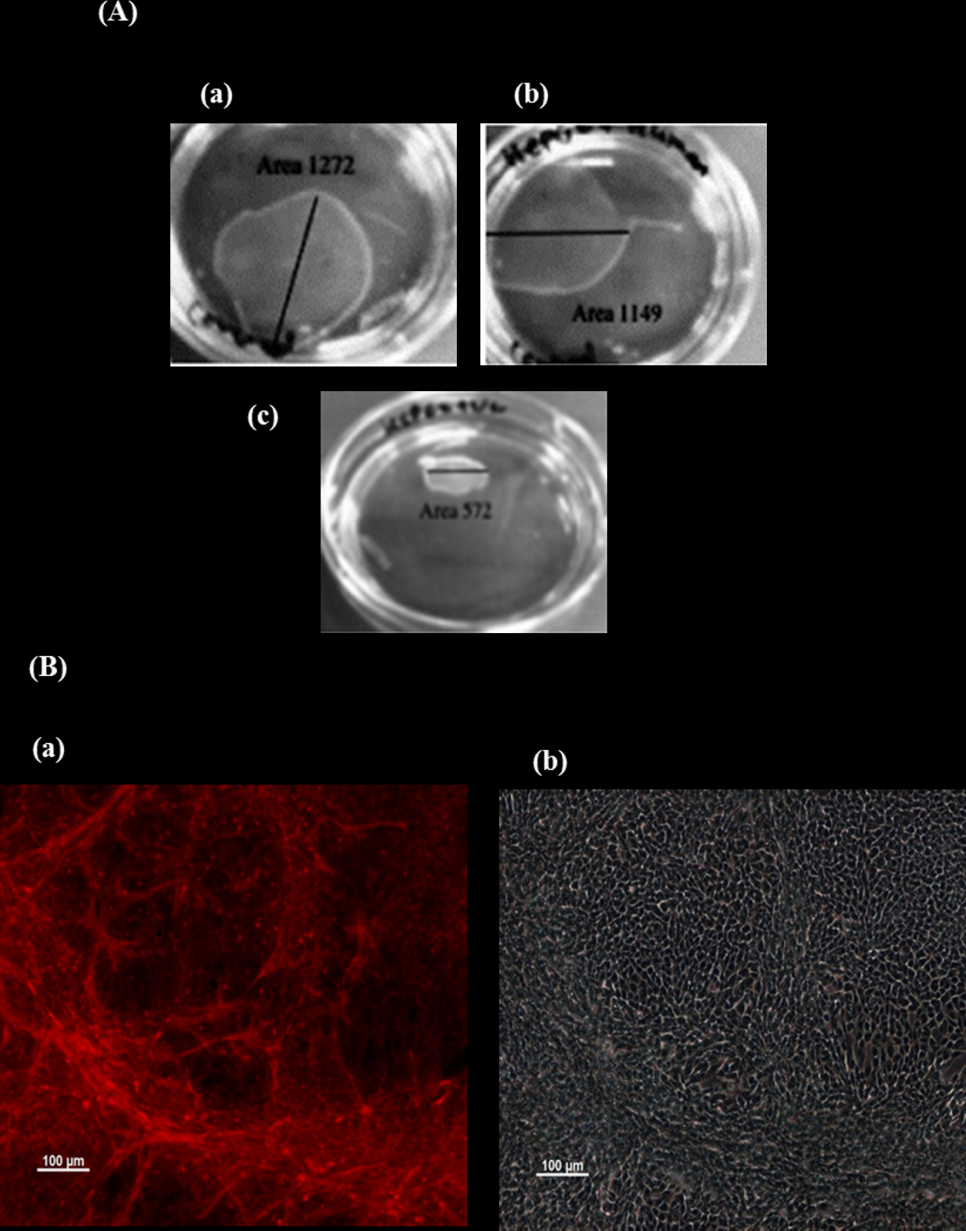
Fabrication of cell sheets using UPcell dishes. (A): (a); HepG2, (b); co-culture of HepG2+BM-MSCs, (c); HepG2+UC-MSC. The Mean of the total surface areas of HepG2, HepG2+human UC-SCs and HepG2+human BM-MSCs; 20 mm 2, 10 mm 2, and 19.8 mm 2 respectively. (B); Fluorescent staining of F-actin filaments in fixed cell sheet of HepG2 and stromal cells. Where (a); HepG2 and UC-SC, and (b) HepG2 and BM-SC. x100 magnification. Staining of F- actin (red) indicates the denseness of differentiated tube formation network of HepG2 and UC-MSC in pouch-like cell sheets in vitro.

Two types of HepG2 sheets were formed when HepG2 cells were cultured with SCs. The addition of UC-MSCs to HepG2 cells resulted in a shrunken type of sheets while BM-MSCs, or only HepG2 resulted in more extended type of sheets (Fig 6A). The total mean of surface areas of the sheets for only HepG2, HepG2- UC-MSCs, and HepG2- BM-MSCs were 20mm^2^, 10mm^2^, and 19.8 mm^2^, respectively.

F-actin is considered as a driving force of contraction [33], therefore we stained the sheets fabricated from HepG2 co-cultured with UC-MSCs or BM-MSCs with F-actin. HepG2- UC-MSC sheet was stained considerably with fiber actin (Fig 6Ba) while HepG2 –BM-MSC sheets showed no staining (Fig 6Bb). Staining of F-actin shows the organisation of a dense differentiated network of HepG2 –UC-MSC exists in pouch-like cell sheets, however no network could be seen for HepG2 –BM-MSC. (Fig 6B; a and b). Each fabrication was done in triplicate.

### Effect of SCs on the production of albumin and urea in cell sheets

**Fig 7:**
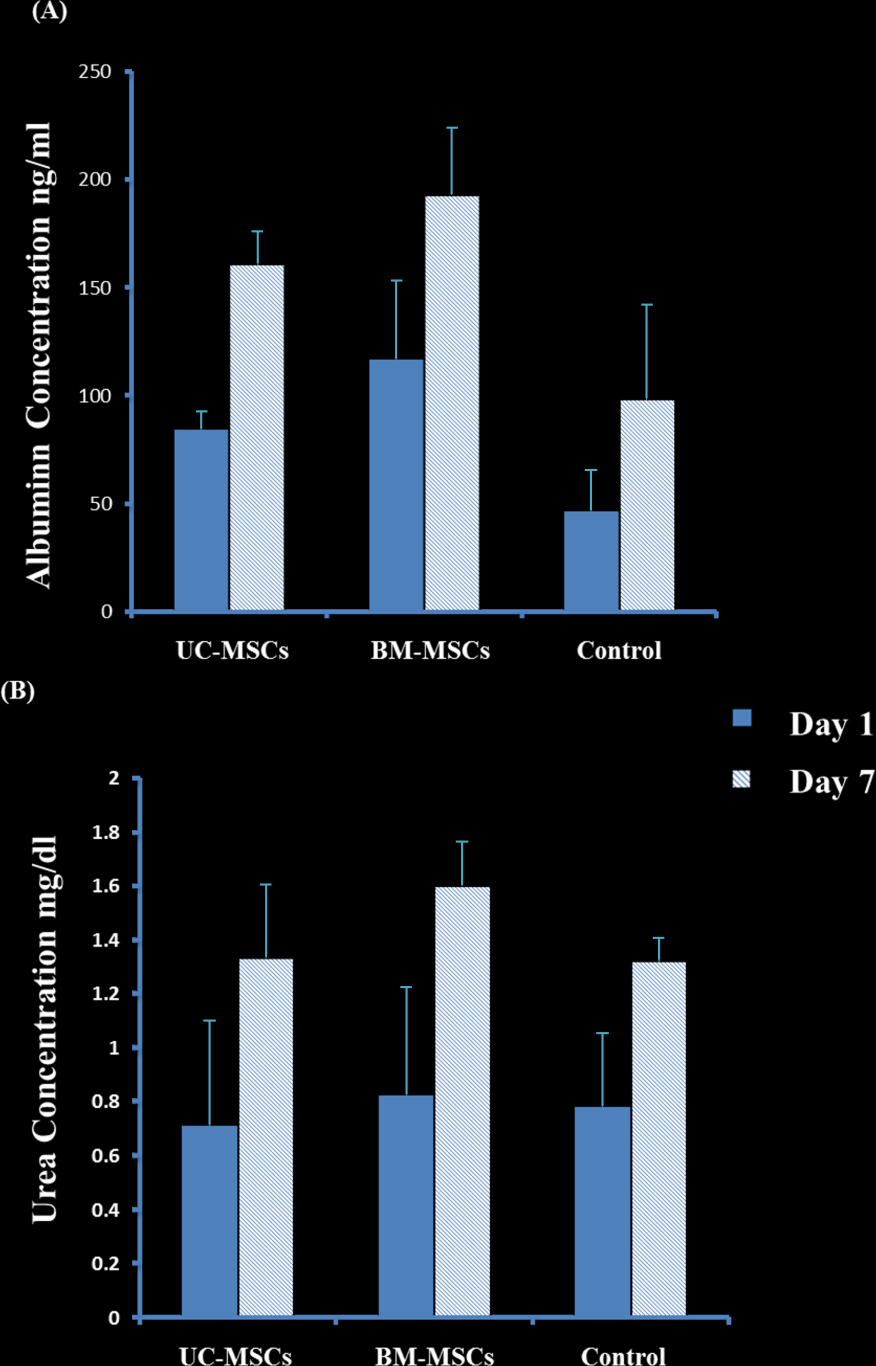
Urea and albumin concentration of HepG2 and stromal cell sheets at day 1 and day 7. The cells were co-cultivated in up-cell dish to form a cell sheet. Where the conditioned medium was collected and HepG2 only cell sheet as control, whereas each stromal cells bar represents co-culture of HepG2 and stromal cells. Albumin or urea secretion by HepG2—UCMSCs, HepG2—BM-MSCs, and HepG2 alone significantly increased at day 7 in culture as compared to day 1, P = 0.009 and P = 0.0004 respectively. Mean±SD. Two-way ANOVA.

The secretion of albumin by the sheet fabricated from HepG2 –UC-MSCs, HepG2 –BM-MSCs and HepG2 alone significantly increased at day 7 in culture as compared to day 1, P = 0.009 (Fig 7A). The addition of UC-MSCs or BM-MSCs to HepG2 sheets significantly increased albumin secretion at day 7 as compared to untreated HepG2, P<0.05 (Fig 7A). However, there was no statistical difference in albumin secretion between the sheets of HepG2 cultured with UC-MSCs and HepG2 with BMM-SCs, P>0.05 (Fig 7A).

Urea secretion by the sheets of HepG2 –UC-MSCs, HepG2 –BM-MSCs and HepG2 alone also increased significantly at day 7 in culture as compared to day 1, P = 0.0004 (Fig 7B), but was not of statistical significance between the three groups at day 1 or 7, P>0.05 (Fig 7B).

### Tumourigenesity of transplanted cell sheets in rats

**Fig 8:**
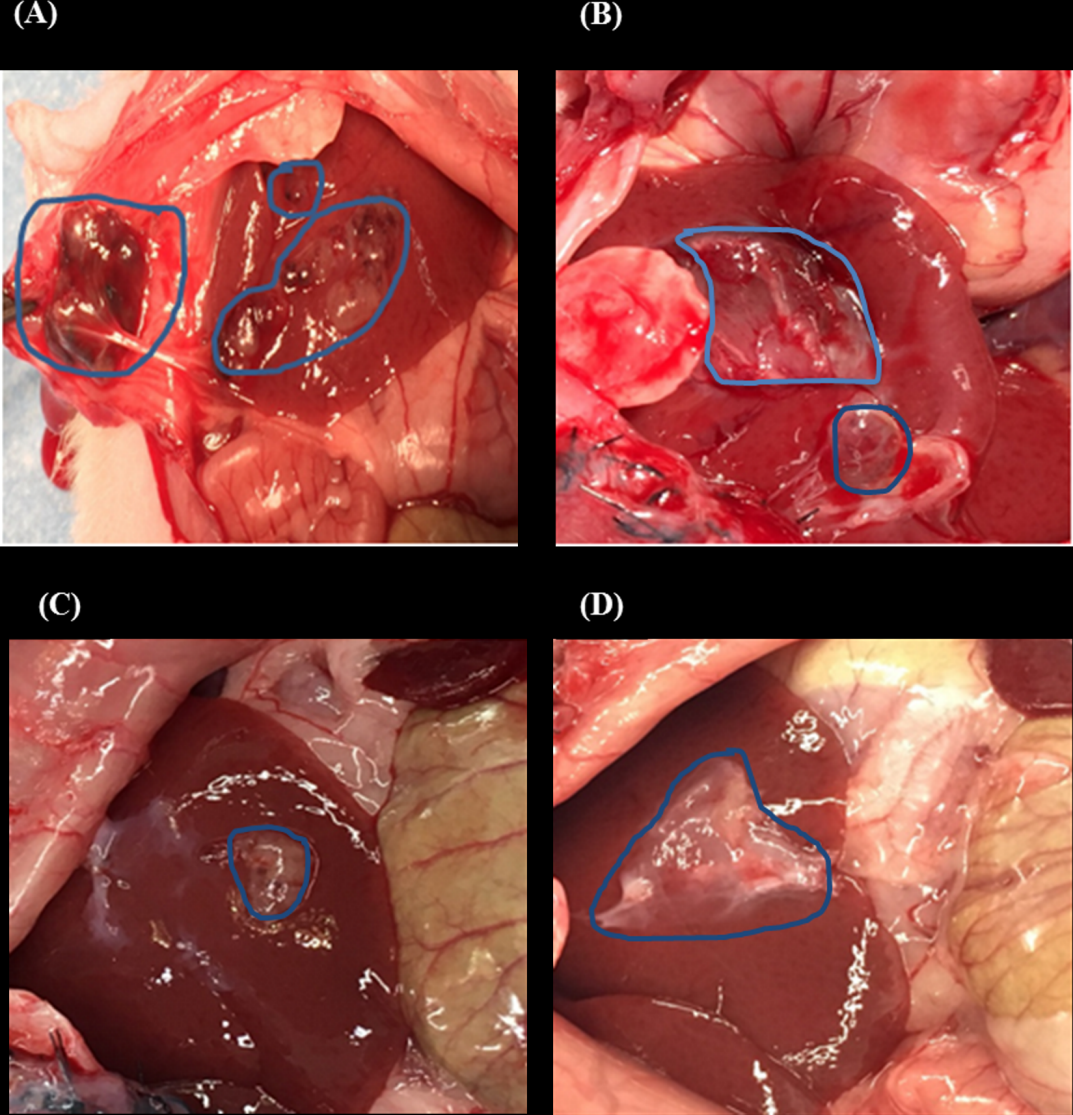
Appearance of rat’s liver after one month after cell sheet transplantation. Where (A) appearance of tumour generated from HepG2 and BM-SC cell sheet, and a mass (2 cm in size) underneath the skin of the rats. (B) cell sheet of HepG2 alone (C) HepG2 and UC-MSC cell sheet, and (D) attached cell sheet of HepG2 and UC-MSC on the liver without forming any tumour. The average size of the tumour developed after transplantation of only HepG2, HepG2 + BM-MSCs, and HepG2+UC-MSC are; 4.5cm, 4cm and 2.5cm respectively, 5 rats was used in each sample group.

After one month of transplantation, rats transplanted with sheets fabricated from HepG2 –UC-MSCs developed no tumours in their liver. All rats transplanted with sheets fabricated from HepG2 and BM-MSCs and from HepG2 alone developed tumours on the surfaces of their livers (Fig 8A and 8B). The average size of the tumours was 4cm and 4.5cm on the liver of rats transplanted with sheets fabricated from HepG2 and BM-MSCs or HepG2 alone, respectively. In contrast, two rats transplanted with UC-MSC and HepG2 had a small sized tumour size that did not exceed 2.5cm (Fig 8C). In addition, a mass of 2cm in size was also observed underneath the skin of rats transplanted with HepG2 and BM-MSCs cell sheet (Fig 8A).

### Histological analysis of liver tissues

**Fig 9:**
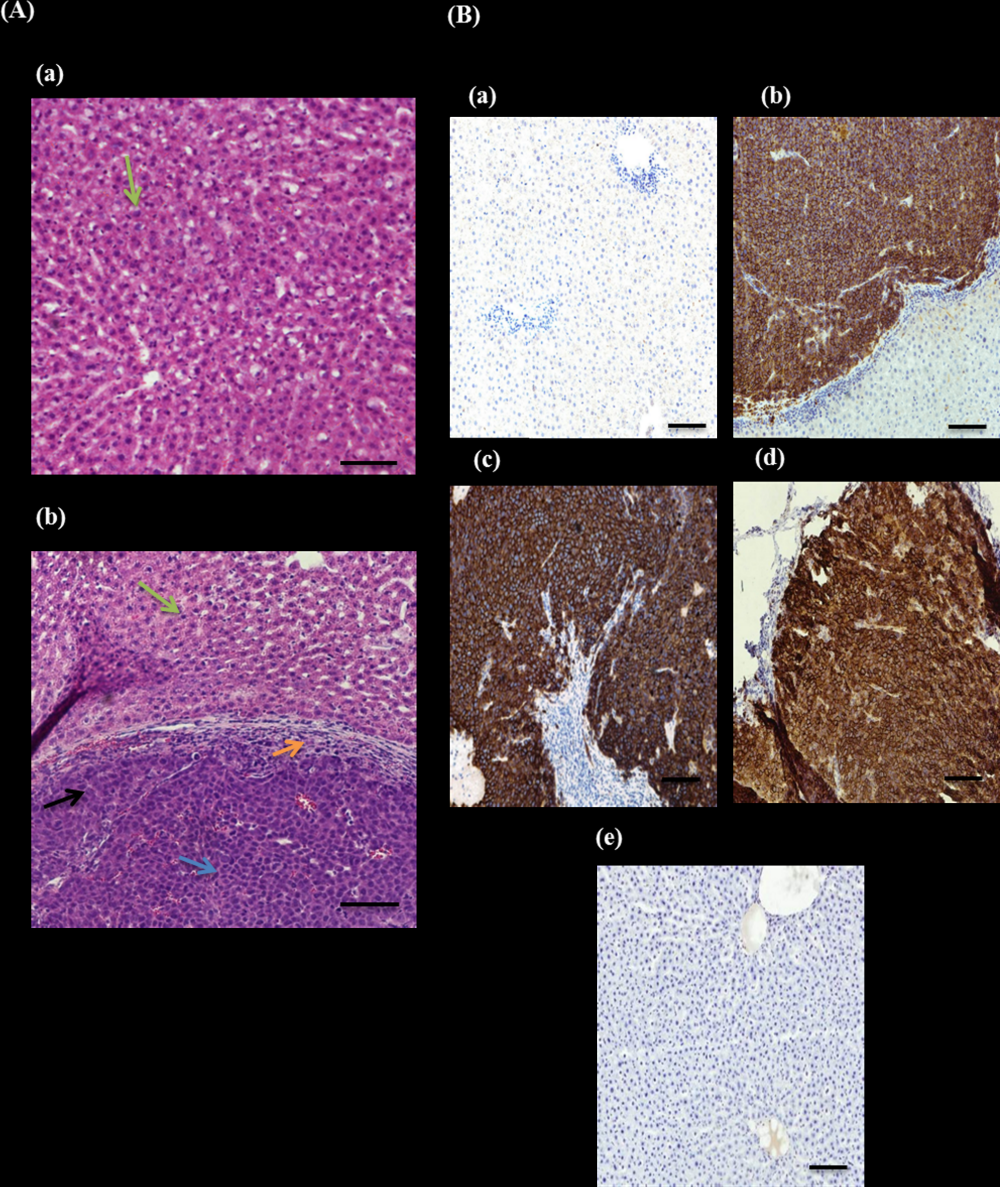
Histological and immunohistochemial analysis of rat’s liver. (A): H&E staining of rat’s liver after 4 weeks of cell sheet transplantation. (a) Normal rat liver cells. (b) Morphology of liver after cancer cells transplantation, black arrow shows hepatic cancer, whereas green arrow shows normal hepatocytes. Blue arrow shows inflamed cell and orange arrow represent necrotic HCC cells, x20 magnification. (B): IHC staining of tumour marker GPC3 antibody on rat’s liver after 4 weeks of transplantation. (a) Negative control, normal rat, no transplantation. (b) HepG2 cell sheet, (c) BM-SC and HepG2 cell sheet on the rat with tumour formation, (d) UC-SC and HepG2 cell sheet on the rat with tumour formation. (e) UC-SC and HepG2 cell sheet on the rat without tumour formation. x10 magnification.

The haematoxylin and eosin (H&E) staining of liver sections revealed that non-transplanted rats and rats transplanted with HepG2 and UC-MSCs sheets had livers with hepatocytes arranged in plates that anastomose with one another and the nuclei were round, with one or two prominent nucleoli (Fig 9Aa). In contrast, livers with tumour from rats transplanted with sheets fabricated from HepG2 –BM-MSCs, HepG2 –UC-MSCs or by HepG2 alone (all of them showed similar morphology) showed edges of viable inflamed and necrotic subcutaneous hepatocytes with irregular nuclear contours and clear degeneration (Fig 9Ab).

### Immunohistochemial staining of liver tissues

The Immunohistochemical staining with an antibody that recognises the early diagnostic tumour marker GPC3 showed that liver tissues from non-transplanted rats and rats transplanted with HepG2 –UC-MSCs sheets had no staining (Fig 9Ba and 9E). In contrast, hepatic cancer cells, from rats transplanted with sheets fabricated from HepG2 –BM-MSCs, HepG2 –UC-MSCs and by HepG2 alone, showed a highly membranous pattern of staining with GPC3 (Fig 9B; b, c and d).

### Effect of SCs on albumin and urea secretion in rats after transplantation

**Fig 10:**
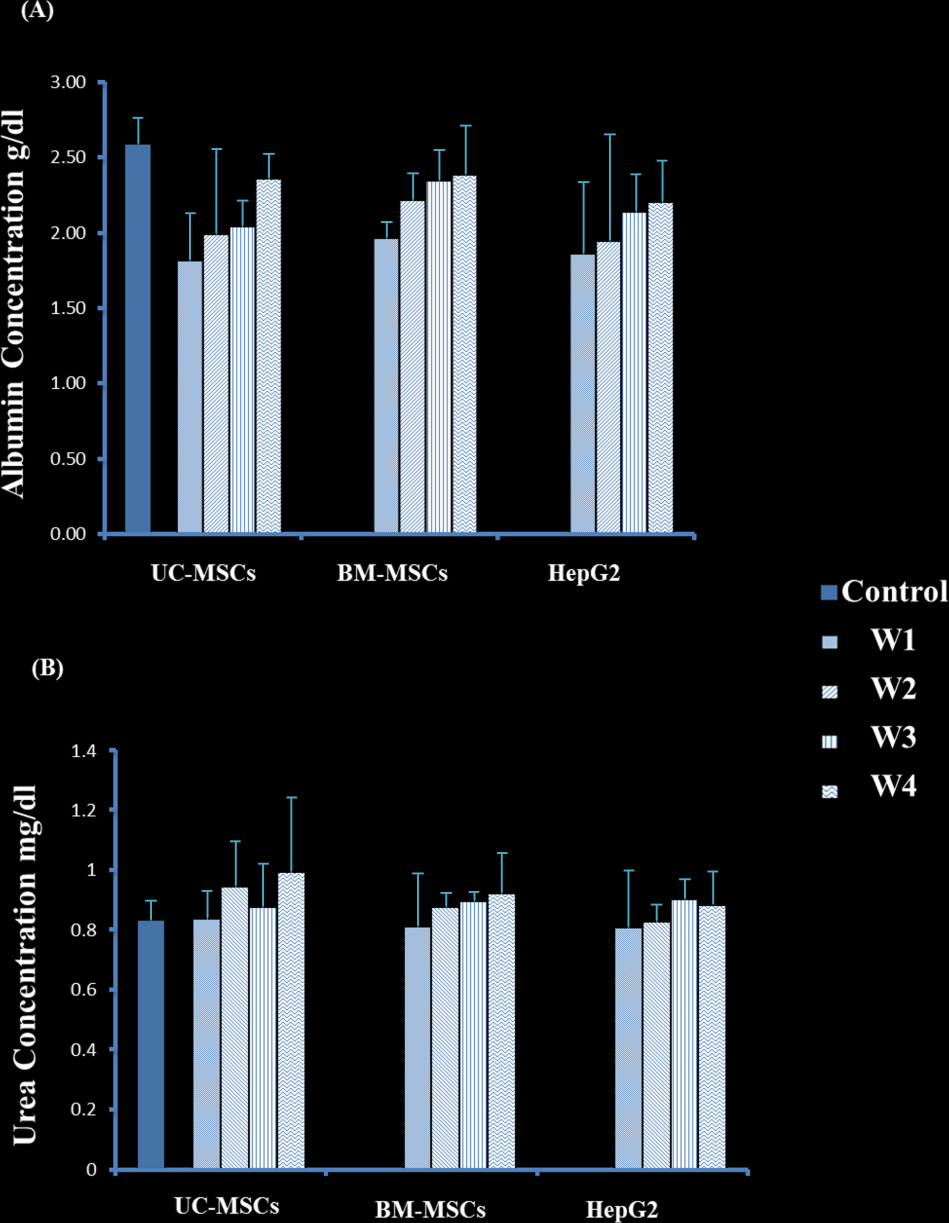
Albumin and urea concentration of rats transplanted with HepG2 and stromal cell sheets within 4 weeks. Where control is non-transplanted rat, whereas each stromal cells bar represents mixed cells of HepG2 and stromal cells. The highest level was detected on week 4. HepG2 and UC-SCs or HepG2 and BM-SCs secreted higher level of albumin as compared to rats transplanted with HepG2 alone, P<0.05). Tow way anova. W = week. Mean±SD.

After 4 weeks, rats transplanted with sheet fabricated from HepG2 and UC-MSCs or HepG2 and BM-MSCs secreted higher level of albumin as compared to rats transplanted with HepG2 alone, P<0.05 (Fig 10A). Within the group of rats transplanted with HepG2 and UC-MSCs, the secretion of albumin after 4 weeks of transplantation is significantly higher than weeks 1, 2 and 3, P<0.05 (Fig 10A) while after 1 week of transplantation, albumin secretion was the lowest, P<0.05 (Fig 10A). Similarly, within the group of rats transplanted with HepG2 and BM-MSCs or with HepG2 alone, albumin secretion was the lowest after 1 week of transplantation, P<0.05, but there was no difference in albumin secretion between weeks 2, 3 and 4, P>0.05 (Fig 10A). In addition, there was no difference in albumin secretion between weeks 1, 2, 3 and 4 in rats transplanted with sheets fabricated from HepG2 and UC-MSCs or HepG2 and BM-MSCs, P>0.05 (Fig 10A).

The secretion of urea in rats transplanted with sheets fabricated from HepG2 and UC-MSCs was higher after 4 weeks of transplantation as compared to weeks 1 and 3 (P<0.05), but at week 2, P>0.05 (Fig 10B). Furthermore, the urea secretion in rats transplanted with sheets fabricated from HepG2 and UC-MSCs was higher than rats transplanted with sheets fabricated from HepG2 and BM-MSCs or HepG2 alone after 4 weeks of transplantation, P<0.05 (Fig 10B). Within the group of rats transplanted with HepG2 –BM-MSCs and HepG2 alone, the secretion of urea was not changed significantly, P>0.05 (Fig 10B).

## Discussion

In this study, we investigated the effect of stromal cells derived from different sources on the development of hepatocellular carcinoma in an animal model. Developing cancer in animals using cell suspension has several limitations, such as poor engraftment due to loss of adhesion molecules during the process of preparation, uncontrolled tumour size, and rapid transition of transplanted cancer cells to the blood circulation, hence, delivery to non-targeted organs [34]. In addition, the infusion of cells through portal vein is associated with a risk of thromboembolism [35]. Moreover, the transplantation of a tumour tissue in animals to develop tumour is also associated with a problem such as the development of a small size of transplanted tumour as a result of death by necrosis due to poor oxygen environment [36]. Furthermore, it has been reported that engineering hepatocytes in a cell sheet can maintain the functions of hepatocyte engineered tissues as compared to the transplantation of isolated hepatocytes [37]. This is due to the creation of appropriate tumour environment and generation of stable and larger tumour size. Therefore, we developed hepatocellular carcinoma in rats using cell sheet technology and we examined the effects of SCs on tumour development and liver parameters secretion. We used hepatocellular carcinoma cell line to construct cell sheets using temperature-responsive UpCell culture dishes as previously published [31]. These HepG2 cells were fabricated with the presence or absence of UC-SMCs or BM-MSCs. The addition of SCs to HepG2 sheets enhanced the fabrication of the sheets as SCs reduced the time to construct and detach the sheets from the culture dishes. Structurally, SCs strengthened HepG2 sheets by reducing their fragility. There was a difference between the types of HepG2 sheets constructed from UC-MSCs and BM-MSCs. The addition of UC-MSCs to HepG2 sheets produced a shrunken type of sheets while BM-MSCs resulted in an extended type of sheets (Fig 6A). This was evident by a reduced total surface area of HepG2 fabricated with UC-MSCs (Fig 6A). In this study, staining of F- actin shows the organisation of a dense differentiated network of HepG2 –UC-MSC, but not HepG2 –BM-MSC, in pouch-like cell sheets. (Fig 6B; a and b). Thus, explaining the shrinking feature of HepG2 –UC-MSCs sheets as a result of reorganisation of cytoskeletal tensile and the contractility functions of F-actin which is known for its ability to drive the contraction of cells [33]. In the present study, HepG2 cells maintained their ability to produce albumin and urea in all fabricated sheets. The production of albumin and urea significantly increased with increasing time in culture (Fig 7), and the addition of SCs to HepG2 sheets enhanced HepG2 secretion of albumin and urea in culture (Fig 7). However, UC-MSCs was more effective in stimulating the secretion of albumin by HepG2 in the cell suspension co-culture system as compared to BM-MSCs (Fig 5). This difference in the results drawn from the cell suspension and sheets may be attributed to the shrunken feature of HepG2- UC-MSC sheet which could reduce the effect of UC-MSCs on the albumin/urea secretion activity of HepG2 in cell sheets.

Using MSCs in cancer therapy is controversial. It has been shown that the transplantation of BM-MSCs or MSCs derived from human adipose tissues with cancer cells into mice stimulates tumour growth by promoting the proliferation and angiogenesis of tumour cells [38, 39]. In addition, it was also reported that the transplantation of adipose-tissue-derived MSCs with glioblastoma multiforme (GBM) cells into mice supported GBM development by promoting angiogenesis and inhibiting apoptosis [25]. In contrast, UC-MSCs transplanted with GBM into mice resulted in a significant reduction in tumour growth [25]. The use of UC-MSCs and BM-MSCs in hepatocellular carcinoma developed in animals is not studied yet. In this study, we developed liver tumour in nude rats by transplanting HepG2 sheets for one month. The development of tumour was completely prevented after the addition of UC-MSCs to HepG2 sheet except in two of the examined rats that developed small sized tumour not exceeding 2.5cm (Fig 8). In contrast, the addition of BM-MSCs to HepG2 sheet did not prevent tumour development in all transplanted rats as liver was inflamed and associated with necrotic cells and degeneration while livers from rats transplanted with HepG2- UC-MSCs sheet showed normal morphological characteristic (Fig 9). Furthermore, livers from rats transplanted with sheets fabricated from HepG2 –BM-MSCs or HepG2 alone showed increased expression of GPC3, the early diagnostic tumour marker while the livers of rats transplanted with HepG2 –UC-MSCs showed no expression of GPC3 (Fig 9). In vitro, UC-MSCs did not form tube network and prevented the HepG2 formation of tube network, unlike BM-MSCs which formed tube network either cultured alone or with HepG2 (Fig 2 and Fig 3). This may refer to the higher secreted level of VEGF from BM-MSCs than UC-MSCs (Fig 4). It is known that new growth in the vascular network is essential for the proliferation and metastatic spread of cancer cells depends on sufficient supply of oxygen and nutrients and the removal of waste products [40]. In addition, it was shown that BM-MSCs can induce gastric carcinoma in a mouse model of chronic Helicobacter infection [41]. Moreover, another study showed BM-MSCs can promote tumour growth in an animal model of breast cancer through promoting tumour cell proliferation and angiogenesis that result in increasing solid tumour growth [42]. This may explain the development of tumours in rats transplanted with HepG2- BM-MSCs. UC-MSCs improved secretion of urea after one month of the transplantation of HepG2 –UC-MSC sheet as compared to BM-MSCs transplanted with HepG2 or HepG2 transplanted alone (Fig 10). This recovery effect of UC-MSCs on the secretion of liver parameters may occur through stimulating the metabolic function of hepatocytes to metabolise ammonia as previously reported [43]. Additionally, after one month of transplantation, UC-MSCs transplanted with HepG2 improved liver secretion of albumin as compared to HepG2 transplanted alone but it was similar to the effect of BM-MSCs on albumin secretion by the liver after transplantation of HepG2- BM-MSCs sheet (Fig 10). This again confirms the ability of UC-MSCs to recover the secretion of liver’s parameters.

The opposite effects of UC-MSCs and BM-MSCs on the development of liver tumour clearly demonstrate the significance of choosing the cell source that can be used safely in patients. Our study shows that UC could be a good source for cells that have a therapeutic potential to modulate liver cancer. It is possible that UC-MSCs may function to prevent tumour formation through blocking the formation of blood vessels. However, more preclinical data regarding mechanisms of action, long term safety and efficacy are necessary before using UC-MSCs in patients.

## Conclusion

This is the first study to develop liver tumour in rats using hepatocellular carcinoma sheet. In this study, we have demonstrated for the first time the potential of using cell sheet technology to develop a HCC rat model. Furthermore, we showed the effectiveness of stromal cells from human umbilical cord tissue in preventing liver tumour formation and improving secretion of albumin and urea as parameters of liver function. The findings of this study provide the foundation for future application of MSCs in preclinical and clinical studies.
